# An innovative approach to study human-snake interactions: insights into tourists’ perception of vipers and their ability to detect them

**DOI:** 10.7717/peerj.21420

**Published:** 2026-06-23

**Authors:** Angel Valentinov Dyugmedzhiev

**Affiliations:** Department of Ecosystem Research, Ecological Risk and Conservation Biology, Institute of Biodiversity and Ecosystem Research, Bulgarian Academy of Sciences, Sofia, Bulgaria

**Keywords:** *Vipera ammodytes*, *Vipera berus*, Snake models, Behaviour, Venomous

## Abstract

In many cultures, snakes are perceived in a markedly negative light, causing hostile attitudes, fear and phobias in people, which often leads to their persecution and killing. Studies on human perceptions of snakes are typically survey-based, while people’s ability to detect them is usually conducted under controlled artificial conditions. Studies that simulate natural circumstances of human–snake encounters, however, remain scarce. The present study recreated natural human–snake encounters by conducting *in situ* experiments with viper models and carcasses, in order to assess tourists’ ability to detect vipers (*Vipera berus* and *Vipera ammodytes*) and to evaluate their reactions. Most people did not detect the vipers when they were placed on the tourist paths, and almost none detected vipers positioned in the grass next to the paths. On the paths, adult vipers were detected significantly more often than juveniles. Most tourists showed interest and no signs of fear during their encounters with the vipers, while a very small number of people exhibited strong fear or aggressive behavior. Tourists were more likely to display no interest and moderate fear toward adult vipers than toward juveniles. Most participants were able either to correctly identify the species or at least recognize that the snake was venomous. These results demonstrate that this innovative approach provides valuable insights into different aspects of human–snake interactions and highlight the usefulness of snake models in such studies.

## Introduction

Snakes play a key role in their ecosystems—they are predators that act as natural regulators of the number and density of rodent and reptile populations, while at the same time serve as food for many species of birds and other small predators (Vitt & Caldwell, 2014). Climate change as well as different human activities (*i.e.,* habitat destruction, intentional killing, overexploitation, illegal trade, *etc.*) have led to a worldwide decline in snake populations (Gibbons et al., 2000; Webb & Shine, 2000; Reading et al., 2010). Consequently, this may negatively impact populations’ natural dynamics which might potentially cause a trophic cascade that leads to deterioration of the ecosystems (Pandey et al., 2016).

A venomous snake’s bite may cause serious damage and even death in humans (Gold, Dart & Barish, 2002; Kasturiratne et al., 2008). This contributes to their negative image in most human societies, and historically they have been subjected to strong hostile attitudes, fear and phobias, which often lead to their persecution and killing (Landová et al., 2012; Landová et al., 2018; Landová et al., 2020; Alves et al., 2014; García-López, Pacheco-Coronel & Gómez-Álvarez, 2017; Eid et al., 2021; Da Silva et al., 2021; Čubrić & Crnobrnja-Isailović, 2022). Although only 15% of snake species are venomous (Gold, Dart & Barish, 2002), the negative attitudes are usually directed towards the entire group as many people are unable to distinguish between venomous and non-venomous species (Alves et al., 2014; Pandey et al., 2016; García-López, Pacheco-Coronel & Gómez-Álvarez, 2017; Valkonen et al., 2018; Wolfe, Fleming & Bateman, 2020; Eid et al., 2021; Kontsiotis, Rapti & Liordos, 2022; Pradhan & Yonle, 2021). Such negative perceptions pose a significant threat to snake conservation, making snakes among the most difficult and unpopular animals to protect and thereby undermining conservation efforts (Alves et al., 2014; García-López, Pacheco-Coronel & Gómez-Álvarez, 2017). Therefore, it is important that snake conservation strategies consider the interactions and perceptions of the local people (Alves et al., 2014; Pandey et al., 2016; Kontsiotis, Rapti & Liordos, 2022).

Studies on human attitudes towards snakes have been carried out in many countries (Landová et al., 2012; Landová et al., 2018; Landová et al., 2020; Ballouard et al., 2013; Alves et al., 2014; Keener-Eck, Morzillo & Christoffel, 2020; Da Silva et al., 2021; Eid et al., 2021; Čubrić & Crnobrnja-Isailović, 2022; Kontsiotis, Rapti & Liordos, 2022). Usually, they are conducted only on a survey basis, where scientists ask a series of questions to local people and, based on the answers received, analyze their attitude towards snakes. However, this method might have some shortcomings and limitations. For instance, participants’ responses may not always be entirely sincere due to discomfort or concerns related to the presence of researchers, especially in face-to-face surveys. These concerns might be even more valid when surveying the participant’s willingness to persecute or kill snakes. Since most snakes are protected by law in many countries, and their collection, persecution or killing is illegal, participants might hesitate to give honest answers, due to the fear of legal consequences. In some instances, human attitude and fear towards different snake species were evaluated *via* experiments, using live snakes and/or their pictures, which were presented to the participants in a controlled environment (Landová et al., 2012; Landová et al., 2018; Landová et al., 2020). However, studies simulating the natural circumstances of a human-snake encounter would be much more beneficial, as they can detect the primal and sincere involuntary (and often subconscious) participants’ reactions. However, to date such studies are lacking, at least to the author’s knowledge.

Because snakes have been a serious threat to primates and humans throughout their evolution, and bites by venomous snakes still cause significant mortality and risks to people’s health (Soares et al., 2014), it has been suggested that primates and humans may have evolved a specialized visual system for rapid snake detection (Öhman, Flykt & Esteves, 2001; Van Strien, Franken & Huijding, 2014). According to Isbell’s snake detection theory (Isbell, 2006), the need for rapid detection and thus avoidance of snakes had a major impact on the evolution of primates’ and humans’ visual system. Multiple studies give support to this theory by providing evidence of enhanced and prioritized human detection of snakes, compared to other non-threatening objects (Öhman, Flykt & Esteves, 2001; LoBue & De Loache, 2008; LoBue & De Loache, 2011; Soares et al., 2014; Van Strien, Franken & Huijding, 2014; Van Strien & Isbell, 2017; Jensen & Caine, 2021), although see also Zsido et al. (2024). Almost all of these studies, however, are conducted under controlled artificial conditions, where schematic drawings, images, or pictures of snakes are presented to the participants on a computer screen in the laboratory. Furthermore, all of these experiments have participants concentrated on the screen in search of an object or reacting to an event. However, these methods highly deviate from the circumstances in which human-snake encounters usually happen. Therefore, the possible existence of a fear module for snake detection should be confirmed in an ecologically valid context, ideally in natural circumstances (Coelho et al., 2019; Jensen & Caine, 2021). Such circumstances should test participant’s ability to detect snakes in natural environment while their attention is directed elsewhere (*e.g.*, walking, looking around, interacting with others, *etc.*) (Jensen & Caine, 2021). However, to date only few studies, recreating more natural circumstances have been conducted (Albergoni et al., 2016; Valkonen et al., 2020; Jensen & Caine, 2021; Lock & Griffiths, 2022).

The use of snake models is a method often used in ecological and conservation studies of snakes. This method is usually used to determine the thermoregulatory abilities of snakes (Bakken, Santee & Erskine, 1985), or the vulnerability of a given species to various predators, such as birds, mammals, *etc.* (Andrén, 1985; Bittner, 2003; Niskanen & Mappes, 2005; Valkonen, Nokelainen & Mappes, 2011; Valkonen et al., 2011; Bateman, Fleming & Wolfe, 2017; Duchesne et al., 2022; Banci et al., 2024; Móré et al., 2024). However, at least to the author’s knowledge, this method has not been tested in studies on people’s reactions to snakes. In addition, snake models have been used in only few studies on people’s ability to detect snakes (Albergoni et al., 2016; Valkonen et al., 2020; Jensen & Caine, 2021; Lock & Griffiths, 2022). The study of Jensen & Caine (2021) was based on a virtual simulation, rather than on natural *in situ* design. In the other three studies, the experiments were conducted in an entirely natural settings, however, the appearance of the models were shown to the participants prior to the experiments, and they were tasked to find as many of them as they can in the field. Furthermore, the models used in the study of Albergoni et al. (2016) were very conspicuously colored and unrealistic, not resembling any particular species.

Viperid snakes are widely distributed on all continents, except Australia and Antarctica, and they are responsible for most fatal venomous snakebites worldwide (Landová et al., 2020). Their distinct morphology makes them easily recognizable and they are known to elicit strong fear in humans (Landová et al., 2018; Landová et al., 2020). All of this makes them an excellent model group for studying the various patterns of human-snake interactions.

There are two species of venomous snakes in Bulgaria, both representatives of the Viperidae family, whose bite could pose a danger to human health—the Balkan Adder *Vipera berus bosniensis* Boettger 1889 and the Nose-horned Viper *Vipera ammodytes* (Linnaeus, 1758) (Stojanov, Tzankov & Naumov, 2011; Tzankov et al., 2014). The Balkan Adder is an endemic subspecies, distributed only on the Balkan Peninsula (Ursenbacher et al., 2006). The Nose-horned Viper is distributed in the southern parts of Central and Eastern Europe and parts of Asia Minor to the Lesser Caucasus (Speybroeck et al., 2016). In Bulgaria, *V. berus bosniensis* inhabits the mid- and high-mountain areas from 800 to 2,700 m a.s.l., (although some lower elevation populations are also known), while *V. ammodytes* is distributed throughout most of the country, except in the high mountains, intensively cultivated agricultural, and urbanized land (Stojanov, Tzankov & Naumov, 2011; Tzankov et al., 2014). While the latter species is protected by Bulgaria’s Biodiversity Act, the former is not (Tzankov et al., 2014). Both species, however, (as well as all other snake species) are generally disliked and often killed on sight by local people (Tzankov et al., 2014; A Dyugmedzhiev, pers. obs., 2025). In addition to killing, populations of *V. ammodytes* in the country have also been subject to intensive exploitation in the recent past, being mass harvested for snake farms for the production of anti-venom serum (Tzankov & Petrov, 2009; Tzankov et al., 2014). However, to date, no studies have evaluated people’s attitudes and reactions toward vipers in Bulgaria, nor their ability to detect them. Such research would support species conservation by providing important scientific and practical insights for understanding and mitigating human–snake conflict, thereby reducing risks to both vipers and humans.

In the current study, the author designed and tested a novel approach to study the different aspects of human–snake interactions. The aim was to recreate natural human–viper encounters as closely as possible by conducting *in situ* experiments with viper models and carcasses, without participants being aware of their involvement. The study assessed tourists’ ability to detect vipers on and next to tourist paths, as well as their reactions upon encountering them. The following hypotheses were tested: (1) tourists notice vipers that are on the path, but not those next to it; (2) tourists notice larger vipers much more often than smaller ones; (3) tourists show fear and often aggressive behavior when meeting vipers; (4) tourists’ reactions to larger vipers are stronger and/or more aggressive than to smaller ones; (5) most tourists are unable to correctly identify the two viper species.

## Materials & Methods

### Study sites

Experiments were conducted *in situ* in two study sites, one for each viper species. Site 1, inhabited by the Balkan Adder *Vipera berus bosniensis*, is located in the Platoto area, Vitosha Mtn., near Sofia city, western Bulgaria (42°36′N; 23°16′E; 1,750–1,850 m a.s.l.). It is an extensive ridge terrace situated above the tree line, covered with alpine and boreal ericoid vegetation, temporary marshes with floating, moving peat, subarctic shrubs of *Salix* spp., and extensive areas covered by Siberian Juniper *Juniperus sibirica*. Site 2, inhabited by the Nose-horned Viper *Vipera ammodytes*, is located near Gara Lakatnik Village, north-western Bulgaria (43°5′N; 23°23′E; 352–733 m a.s.l.). It is a karst valley along the Iskar River, with steep rock cliffs, covered with grass, shrubs and patches of deciduous forests. These exact sites were chosen due to the following criteria: (1) the respective viper species have abundant populations and there are high encounter rates there (Stoyanov & Tzankov, 2017; Dyugmedzhiev et al., 2020); (2) they are popular tourist destinations, where hundreds of tourists per day hike along the few recognizable trails, especially during weekends and holidays (Stojanov, 2014; A Dyugmedzhiev, 2025, unpublished data); (3) these trails pass through vipers’ habitat and, as part of their natural home range, vipers often cross them, or bask and hunt right next to them (A Stojanov & A Dyugmedzhiev, pers. obs., 2016).

### Study design and experiments

**Figure 1:**
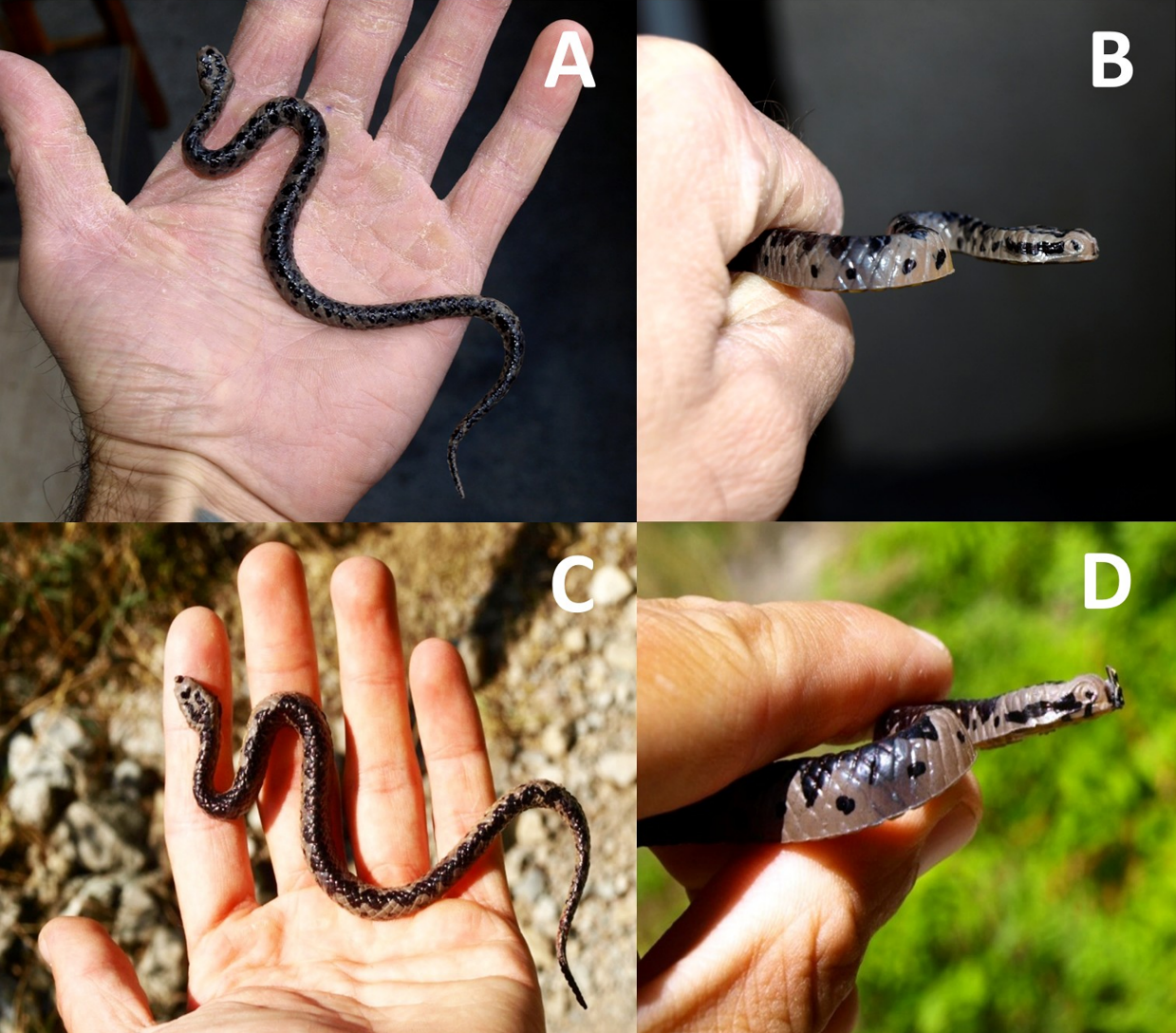
View of the plastic models of juvenile *V. berus* (A and B) and *V. ammodytes* (C and D), used in the experiments.

The study aimed to recreate natural human–viper encounters as closely as possible, without the participants being aware of their involvement. For this purpose, a series of *in situ* experiments were conducted. For the first set of experiments, preserved carcasses of adult females of both *V. berus* (total length of 55.5 cm) and *V. ammodytes* (total length of 56 cm) were used. The individuals were previously collected dead specimens (*i.e.,* found dead on the road or killed by local people), and no animals were killed or harmed for the purpose of the research. Due to the lack of preserved carcasses of juvenile vipers, plastic snake models (total length of 20 cm) were used for the second set of experiments. These models, which were of a uniform brown coloration, were additionally painted and modified in such a way that they resemble a juvenile viper as close as possible. For this purpose, the characteristic species-specific dorsal ziz-zag line, head markings and additional body markings were painted (Fig. 1). In addition, a small “horn” was attached to the snout of the model of *V. ammodytes* for maximum credibility (Fig. 1D). Adult female instead of male carcasses were chosen to avoid bias based on differences in body coloration (Valkonen et al., 2020), as females have similar brown body coloration as the models, in contrast to males, which are more greyish, especially during some parts of the activity period (Speybroeck et al., 2016).

**Figure 2:**
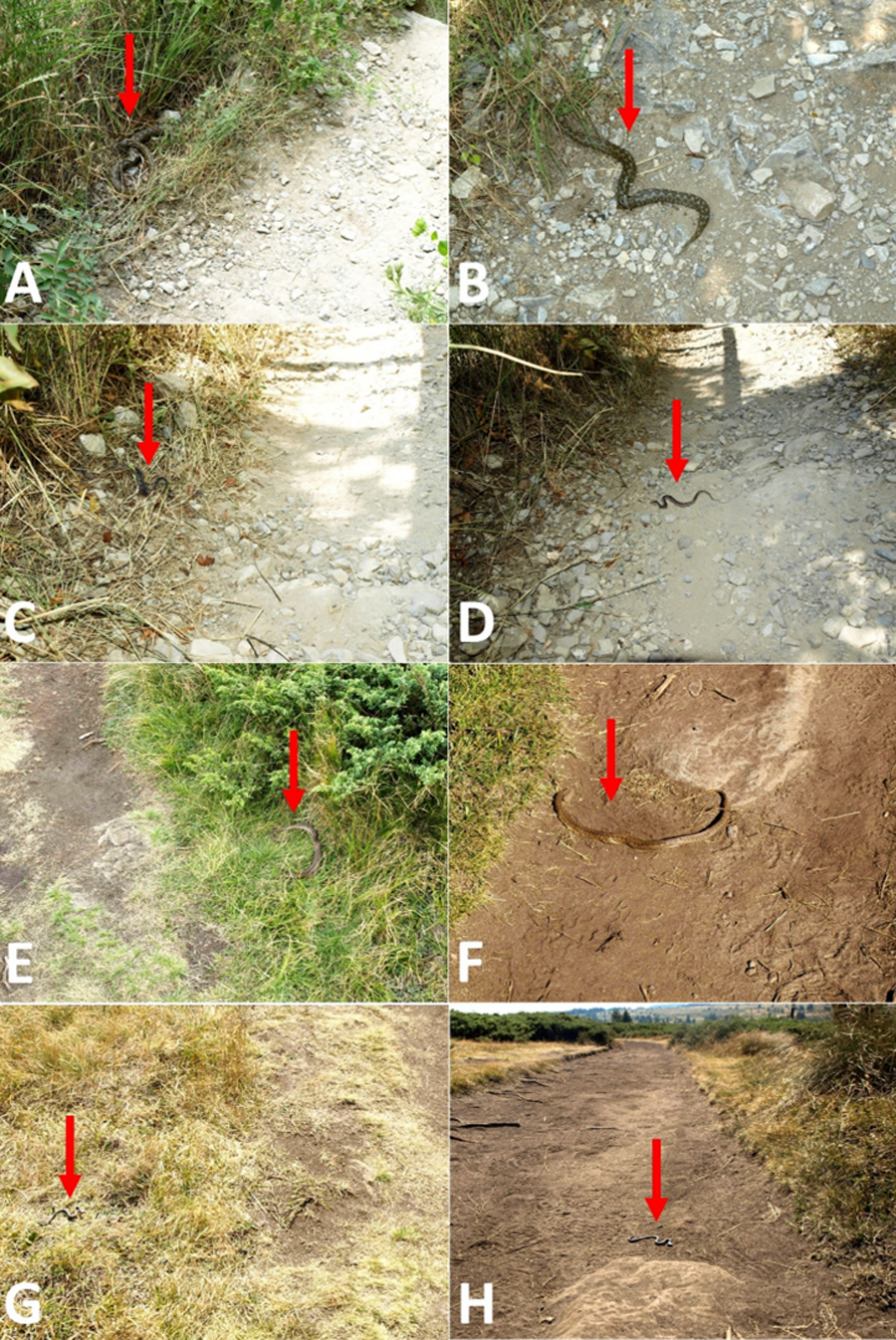
View of the placement of the viper carcasses/models during the experiments. (A and C) Placement next to the path of the adult carcass and the juvenile model of *V. ammodytes*, respectively; (B and D) placement on the path of the adult carcass and the juvenile model of *V. ammodytes*, respectively; (E and G) placement next to the path of the adult carcass and the juvenile model of *V. berus*, respectively; (F and H) placement on the path of the adult carcass and the juvenile model of *V. berus*, respectively.

All experiments were conducted over the course of 12 different days, on weekends between June and September 2025, within the activity period of both species (Stojanov, Tzankov & Naumov, 2011). At each site, experiments were carried out only with the respective species inhabiting it, including both adults and juveniles. In the first sets, models/carcasses were placed in the grass, just next to the trail, in a position resembling a basking viper (Fig. 2). In the second sets, they were placed on the trail, close to one of its edges, in a position resembling a viper moving away from the trail (Fig. 2). Each set of experiments was conducted separately for adults and juveniles, on different days. To ensure repeatability and comparability, vipers were placed at the same exact location each time (either on or next to the path), and this location was consistent across experiments with both adults and juveniles. Tourists’ behavior and reactions were observed by the author from a few meters away, without any interaction with the participants, thereby avoiding direct interference in the experimental setup. This approach allowed the observation of authentic responses to vipers, as reactions were not influenced by factors such as observer bias or survey-related anxiety. The first line of information in each respective set of experiments was whether the tourist or group of tourists detected the viper while walking on their hike. When tourists detected the viper, each of their respective reactions towards it was described in detail within a special field form. When possible (*i.e.,* tourists discussing it between them), the following additional information was collected: (1) whether they were able to identify the respective species correctly or not; (2) whether they understood that the viper is not alive; (3) whether they understood that the juvenile’s model is not a real viper. Information about detection of the vipers was used only for the tourists that found it themselves, rather than if it were shown to them by someone else. Such cases, included the following situations: (1) a tourist/tourists, walking in front of, or next to others, detected the viper and then showed it to the others, before the latter would have the chance to “find” it themselves; (2) tourists’ dogs, walking few meters in front of them, started sniffing on the viper, and thus showing it to their owners before they would have the chance to “find” it. However, the reactions, as well as each additional information, for all people, that saw the viper, either by themselves or if it was shown to them, were recorded and analyzed. The Institute of Biodiversity and Ecosystem Research Ethics Committee approved the animal study protocol under project “KΠ-06-M81/2 from 02.12.2024, with protocol code 001/14.01.2026. All of the work was carried in accordance with Ministry of Environment and Water of Bulgaria Permit No. 1084/27.02.2025.

### Data processing and statistical analyses

Based on their specifics, the detailed descriptions of each tourist’s reaction towards the vipers were summarized into 20 separate more general reaction categories (Table 1). These 20 categories were then further grouped into several more specific and basic categories. These categorizations were made in regard to three separate behavioral aspects—FEAR, INTEREST and FEAR + INTEREST. FEAR consisted of three basic categories: (1) Strong fear—combining reaction categories 4, 5, 6 and 7; (2) Moderate fear—combining reaction categories 1, 2, 3, 8, 9, 10, 11 and 12; 3) No fear—combining reaction categories 13, 14, 15, 16, 17, 18, 19 and 20 (see Table 1). INTEREST consisted of two basic categories: (1) No interest—combining reaction categories 2, 4, 5, 8, 9, 13, 14, 15 and 19; (2) Interest - combining reaction categories 1, 3, 6, 7, 10, 11, 12, 16, 17, 18 and 20 (Table 1). FEAR + INTEREST combined both aspects and consisted of six basic categories: (1) Strong fear with no interest—combining reaction categories 4 and 5; (2) Strong fear with interest—combining reaction categories 6 and 7; (3) Moderate fear with no interest—combining reaction categories 2, 8 and 9; (4) Moderate fear with interest—combining reaction categories 1, 3, 10, 11 and 12; (5) No fear and no interest—combining reaction categories 13, 14, 15 and 19; (6) Interest with no fear—combining reaction categories 16, 17, 18 and 20 (Table 1). In this way both the independent effect of each of the two main aspects of tourists’ attitude towards the vipers (*i.e.,* the fear and the interest towards them) as well as their combined effect could be evaluated.

**Table 1:** Types and numbers of registered tourists’ reactions towards adult and juvenile *Vipera berus* and *Vipera ammodytes*.

Reaction	*V. berus*		*V. ammodytes*	
	Ad	Juv	Ad	Juv
1. Direct aggression (try to kill it by jumping on its head) with moderate fear (stop abruptly; jump; pull slightly to the side) and interest (stop to look at it; observe it from close distance; photograph it)	0	0	0	1
2. Verbal hostility/dislike (talking about killing it, without really trying; glad it is dead) with moderate fear (stop abruptly; jump; pull slightly to the side) and no interest (passing it by, without stopping)	1	0	0	1
3. Verbal hostility/dislike (talking about killing it, without really trying; glad it is dead) with moderate fear (stop abruptly; jump; pull slightly to the side) and interest (stop to look at it; observe it from close distance; photograph it)	1	0	0	0
4. Very strong fear (turn around and go back, giving up on their hike)	0	0	2	0
5. Strong fear (scream / shout; thinking of turning back and give up on their hike, but not doing it) without interest (passing it by, without stopping)	2	0	0	2
6. Strong fear (scream / shout; thinking of turning back and give up on their hike, but not doing it) with interest (stop to look at it; observe it from close distance; photograph it)	2	1	2	0
7. Strong fear (scream / shout; thinking of turning back and give up on their hike, but not doing it) with interest (stop to look at it; photograph it) and thorough inspection (poking it with a stick or a hiking pole; pushing it with their foot; throwing a pebble at it to check if it is alive; taking the corpse in hand; moving the corpse away from the path)	0	1	0	0
8. Moderate fear (stop abruptly; jump; pull slightly to the side) without interest (passing it by, without stopping)	7	1	11	2
9. Moderate fear (stop abruptly; jump; pull slightly to the side) without interest (passing it by, without stopping) and telling the others not to bother or touch it	0	1	0	0
10. Moderate fear (stop abruptly; jump; pull slightly to the side) with interest (stop to look at it; observe it from close distance; photograph it)	7	17	4	1
11. Moderate fear (stop abruptly; jump; pull slightly to the side), with interest (stop to look at it; photograph it) and thorough inspection (poking it with a stick or a hiking pole; pushing it with their foot; throwing a pebble at it to check if it is alive; taking the corpse in hand; moving the corpse away from the path)	2	3	1	3
12. Moderate fear (stop abruptly; jump; pull slightly to the side), with interest (stop to look at it; photograph it) and pity that it is dead	1	0	0	0
13. No interest (passing it by, without stopping)	14	0	6	3
14. No interest (passing it by, without stopping) but acknowledging its presence (showing it to the others; talk about it, while moving away)	2	7	4	5
15. No interest (passing it by, without stopping) and telling the others not to bother or touch it	1	0	0	1
16. Interest (stop to look at it; photograph it) without visible fear	35	21	10	10
17. Interest (stop to look at it; photograph it) without visible fear and thorough inspection (poking it with a stick or a hiking pole; pushing it with their foot; throwing a pebble at it to check if it is alive; taking the corpse in hand; moving the corpse away from the path)	2	12	0	3
18. Interest (stop to look at it; photograph it) without visible fear and pity that it is dead	2	0	0	0
19. Positive reaction (happy that they saw a snake) without interest (passing it by, without stopping) and no visible fear	0	0	0	1
20. Positive reaction (happy that they saw a snake) with interest (stop to look at it; photograph it), no visible fear and a pity that it is dead	1	0	0	0

A *χ*2 test with Yates’ correction was used in each of the analyses. To analyze vipers’ detection by tourists, four different tests were made: (1) comparison between adult vipers’ detection on and next to the path; (2) comparison between juvenile vipers’ detection on and next to the path; (3) comparison between adult and juvenile vipers’ detection on the path; (4) comparison between adult and juvenile vipers’ detection next to the path. To analyze tourists’ attitudes and reactions towards vipers, the derived basic reaction categories for each of the three behavioral aspects were compared between adult and juvenile vipers. For most of the categories containing Strong fear, the *χ*2 test was not performed due to the low number of these reactions. In each analysis, initially the combined data for the two species was used, to increase sample sizes and thus provide higher power to the tests. Then, the data for each species were analyzed separately to evaluate differences between them. The data for tourists’ ability to identify viper species and the other additional information were not analyzed statistically, due to their low sample sizes. All statistical analyses were conducted with Statistica 10.0 (StatSoft Inc, 2011). Statistical significance was accepted at *p* < 0.05.

## Results

### Detection of the vipers

**Figure 3:**
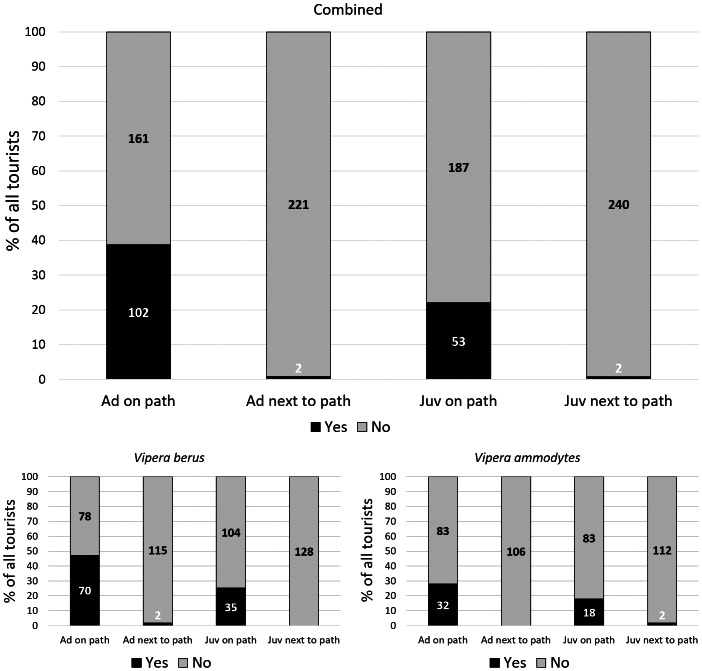
Comparison between tourists’ detection rate of adult and juvenile vipers placed on and next to the path. Numbers within each bar indicate the total number of tourists that detected/did not detected the vipers in the respective tests; Yes indicates cases when tourists detected the viper; No indicates the cases in which they did not detected it. The graph presents the combined data for both species and for each species separately.

A total of 968 tourists were monitored for the analyses on viper detection (532 for *V. berus* and 436 for *V. ammodytes*). Tourists’ detection of both adult and juvenile vipers differed significantly depending on their placement (Table 2), with individuals on the path detected much more frequently than those next to it (Fig. 3). Vipers positioned next to the path were almost never detected. On the path, adult vipers were detected significantly more often than juveniles, whereas no such pattern was observed for vipers located next to the path (Table 2 and Fig. 3). Analyses conducted separately for each species showed similar patterns for *V. berus* (Table 2 and Fig. 3). The same trend was observed for *V. ammodytes*, although the higher detection of adults on the path was not statistically significant (Table 2 and Fig. 3).

**Table 2:** Results from the *χ*2 test analyzing vipers’ detection by tourists and tourists’ reactions to vipers in regards to the three behavioral aspects. DF = 1 in all cases. * marks the cases with statistically significant results; # marks the cases with results, very close to statistical significance.

		Comparison between groups	Yates’ *χ*2	*p*
Detection	Combined	Ad on path *vs* Ad next to path *	100.74	<0.00001
		Juv on path *vs* Juv next to path *	51.78	<0.00001
		Ad on path *vs* Juv on path *	15.64	0.00008
		Ad next to path *vs* Juv next to path	0.18	0.67
	*V. berus*	Ad on path *vs* Ad next to path *	66.34	<0.00001
		Juv on path *vs* Juv next to path *	34.92	<0.00001
		Ad on path *vs* Juv on path *	14.18	0.0002
		Ad next to path *vs* Juv next to path	0.6	0.14
	*V. ammodytes*	Ad on path *vs* Ad next to path *	32.28	<0.00001
		Juv on path *vs* Juv next to path *	14.54	0.0001
		Ad on path *vs* Juv on path	2.49	0.11
		Ad next to path *vs* Juv next to path	0.43	0.51
Fear	Combined	Strong fear: Ad *vs* Juv	0.75	0.39
		Moderate fear: Ad *vs* Juv	0.25	0.62
		No fear: Ad *vs* Juv	1.21	0.27
	*V. berus*	Moderate fear: Ad *vs* Juv	0.09	0.75
		No fear: Ad *vs* Juv	2.64	0.1
	*V. ammodytes*	Moderate fear: Ad *vs* Juv	2.04	0.15
		No fear: Ad *vs* Juv	0.09	0.76
Interest	Combined	No interest: Ad *vs* Juv *	7.68	0.006
		Interest: Ad *vs* Juv	0.007	0.93
	*V. berus*	No interest: Ad *vs* Juv *	8.76	0.003
		Interest: Ad *vs* Juv	0.009	0.92
	*V. ammodytes*	No interest: Ad *vs* Juv	0.92	0.34
		Interest: Ad *vs* Juv	0.03	0.86
Fear+Interest	Combined	Moderate fear with no interest: Ad *vs* Juv *	7.04	0.008
		Moderate fear with interest: Ad *vs* Juv	1.56	0.21
		No fear and no interest: Ad *vs* Juv	1.84	0.17
		Interest with no fear: Ad *vs* Juv	0.09	0.76
	*V. berus*	Moderate fear with no interest: Ad *vs* Juv	2.5	0.11
		Moderate fear with interest: Ad *vs* Juv	2.07	0.15
		No fear and no interest: Ad *vs* Juv #	3.38	0.07
		Interest with no fear: Ad *vs* Juv	0.49	0.48
	*V. ammodytes*	Moderate fear with no interest: Ad *vs* Juv #	3.5	0.06
		Moderate fear with interest: Ad *vs* Juv	0.1	0.75
		No fear and no interest: Ad *vs* Juv	0.05	0.82
		Interest with no fear: Ad *vs* Juv	0.17	0.68

Tourists would often step just next to the viper’s model/carcass on the path or cross over it without contact. In most such cases, people did not detect the viper. For instance, 20 people stepped just next to, or crossed over the adult *V. berus* carcass without seeing it, 12 saw it after they had stepped next to/crossed over it, and five saw it just before they stepped on it. Thirty-two people stepped just next to or crossed over the juvenile *V. berus* model without seeing it (three of them walking barefoot), three saw it after they had stepped next to/crossed over it, one saw it just after it had already stepped on it, and three stepped on it without detecting it at all. In similar pattern, 16 people stepped just next to, or crossed over the adult *V. ammodytes* carcass without seeing it, three saw it after they had stepped next to/crossed over it, two saw it just before they stepped on it, while two stepped on it without seeing it. Twenty-nine people stepped just next to or over the juvenile *V. ammodytes* model without seeing it, one saw it after it had stepped next to it, and two stepped on it without detecting it.

### Tourists’ reactions towards detected vipers

**Figure 4:**
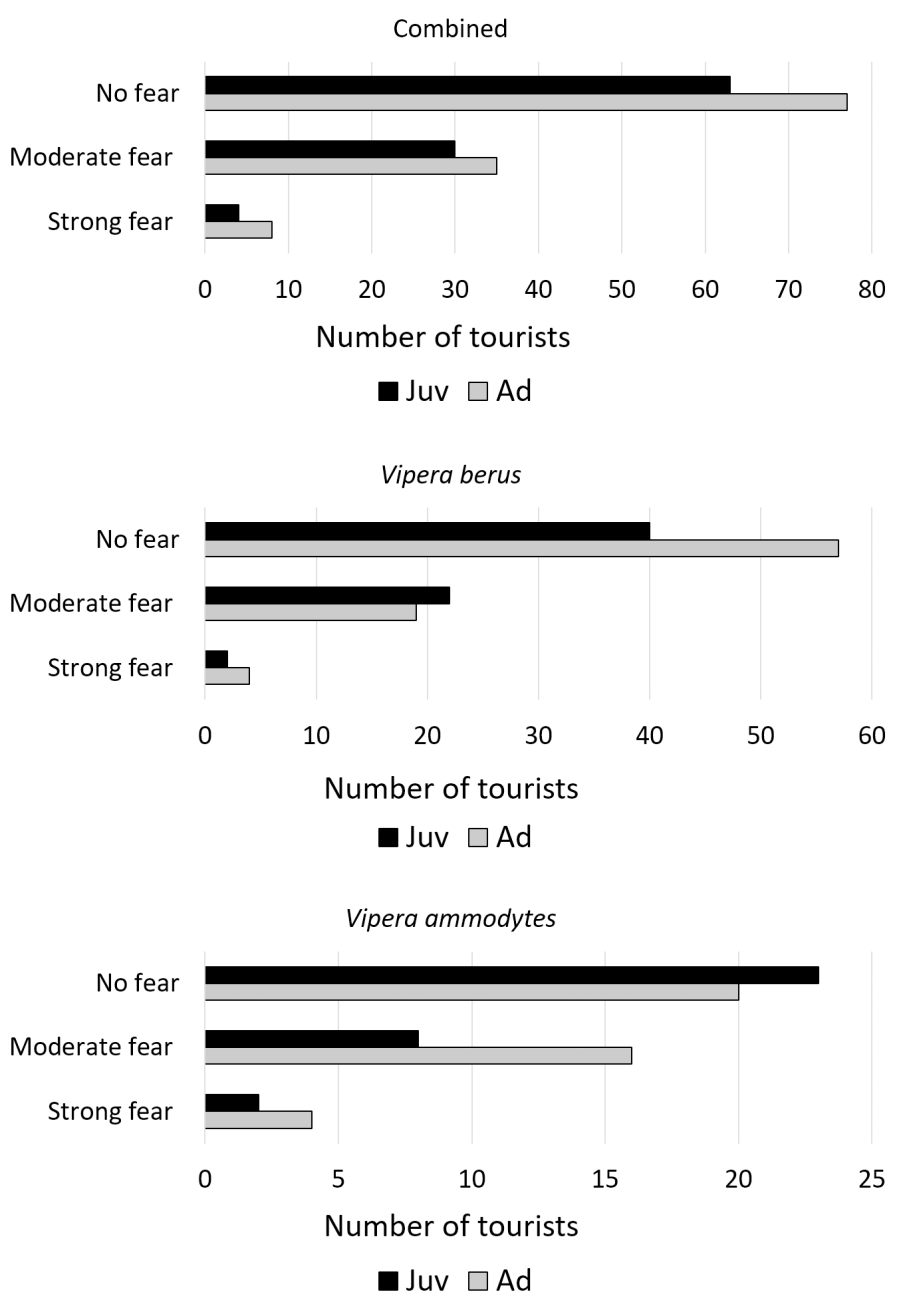
Comparison between tourists’. reactions towards adult (Ad) and juvenile (Juv) vipers in regard to FEAR. The graph presents the combined data for both species and for each species separately.

Reactions from 217 tourists were recorded during the study (for reaction type see Table 1). In regard to FEAR, No fear was the most common reaction, followed by Moderate fear, while Strong fear was very rare. This pattern was clear for both the combined data and for each separate species (Fig. 4). There were no statistically significant differences between the reactions towards adults and juveniles in any of the three categories (Table 2). Although some small differences between adults and juveniles of the separate species were present (*i.e.,* in No fear for *V. berus* and in Moderate fear for *V. ammodytes*, Fig. 4), they were no statistically significant (Table 2).

**Figure 5:**
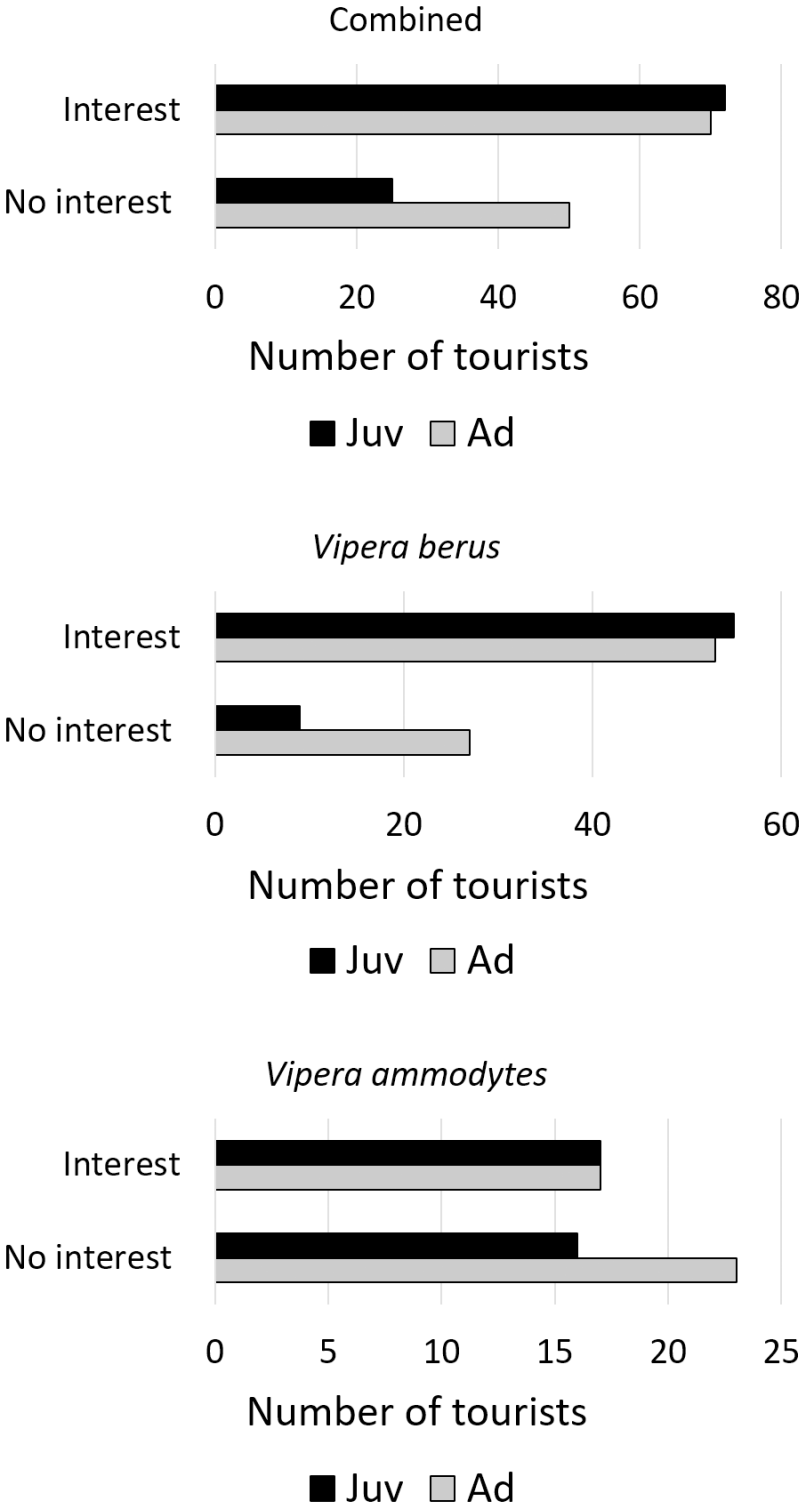
Comparison between tourists’ reactions towards adult (Ad) and juvenile (Juv) vipers in regard to INTEREST. The graph presents the combined data for both species and for each species separately.

In regard to INTEREST, Interest was more common than No interest, except for *V. ammodytes*, where the two reactions were with similar numbers (Fig. 5). Statistically significant differences between the reactions towards adults and juveniles were present only for No interest (Table 2), where tourists tended to show no interest towards adults, more than towards juveniles (Fig. 5). Although this pattern was present for both species (Fig. 5), the results were statistically significant only for *V. berus* (Table 2).

**Figure 6:**
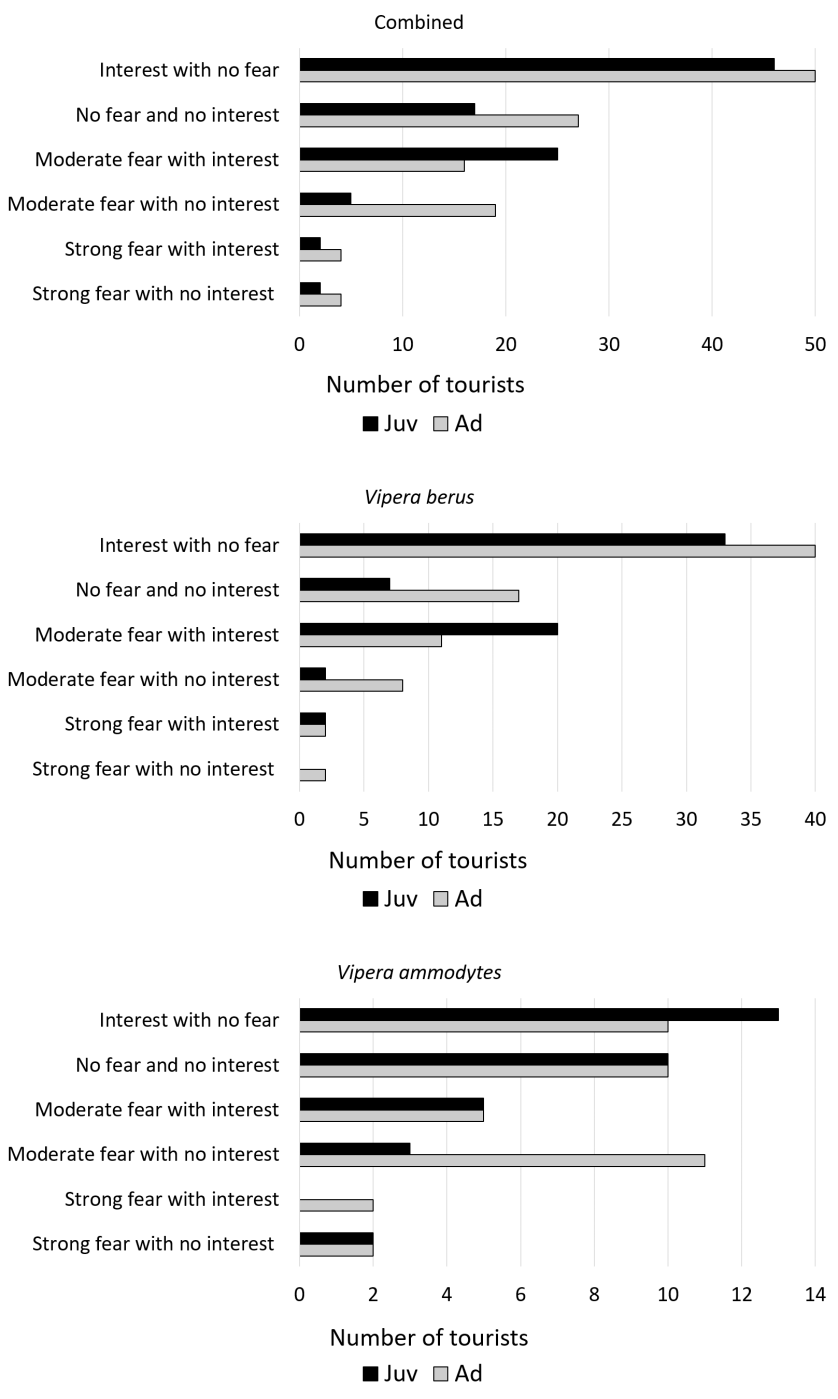
Comparison between tourists’ reactions towards adult (Ad) and juvenile (Juv) vipers in regard to FEAR + INTEREST. The graph presents the combined data for both species and for each species separately.

In regard to the combination FEAR + INTEREST, the most common reaction for the combined data was Interest with no fear, followed by No fear and no interest, Moderate fear with interest and Moderate fear with no interest (Fig. 6) The same overall pattern, with only small deviations, was evident for each separate species (Fig. 6). Statistically significant differences between the reactions towards adults and juveniles were present only for Moderate fear with no interest (Table 2), where this reaction was much more often observed towards adults, than towards juveniles (Fig. 6). When conducting the analyses separately for each species, no statistically significant differences were present; however the results for No fear and no interest for *V. berus* and Moderate fear without interest for *V. ammodytes* were very close to significance (Table 2), with these reactions being more common towards adults (Fig. 6).

### Species identification and additional information

**Figure 7:**
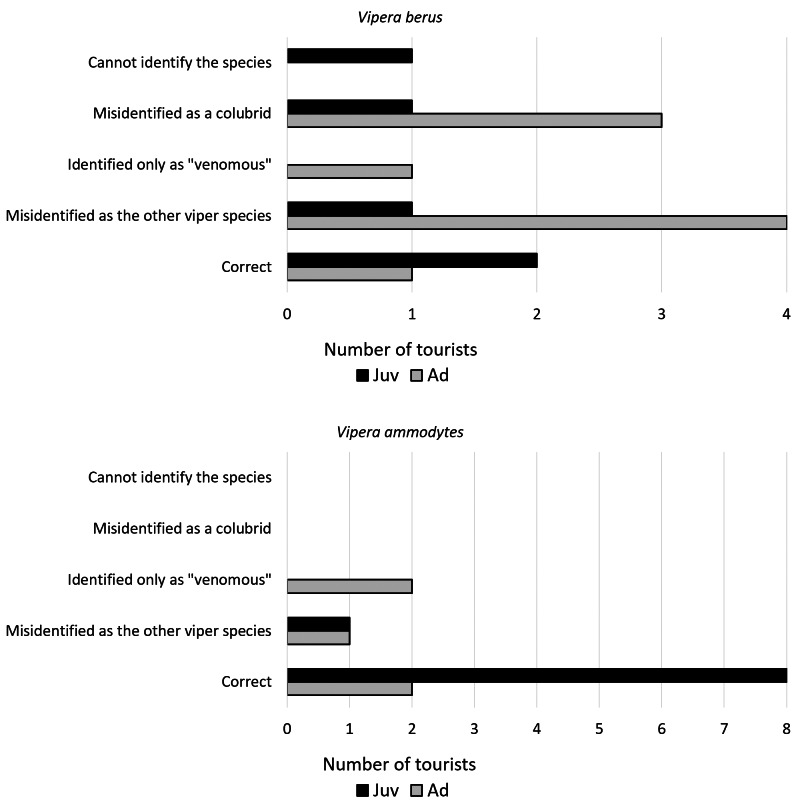
Comparison between tourists’ identification of adult (Ad) and juvenile (Juv) vipers.

In most cases, when a tourist tried to identify the species, the identification was either correct, or the two viper species were misidentified between each other (*i.e., V. berus* identified as *V. ammodytes* and vice versa) (Fig. 7). Most correct identifications, however, were made on the plastic models of *V. ammodytes* juveniles, rather than on the carcasses of adults from both species (Fig. 7). In contrast, carcasses of *V. berus* adults were most often misidentified as *V. ammodytes* or as a colubrid non-venomous snake (Fig. 7).

From the 73 people (of the 97 who reacted to the models), for which such information could be gathered, only 19.18% understood that the models were not real dead vipers, 4.11% were not sure about it, while 76.71% believed that they were real. In all cases where tourists recognized or questioned whether the models were real vipers, this occurred only after they had either carefully observed the model from a very close distance (less than 0.5 m) for at least one minute or, more commonly, after examining it more thoroughly (*e.g.*, poking it with sticks or hiking poles, touching it, or picking it up). In addition, among the 135 individuals (out of 217) for whom such information could be obtained, 98.52% recognized that the vipers were not alive (60% for adults and 38.5% for juveniles), whereas only 1.48% (all for juveniles) did not.

## Discussion

### Detection of the vipers

A critical component of the Isbell’s snake detection theory is that people (and primates) must be able to rapidly detect snakes even when their attention is directed elsewhere (Isbell, 2006; Jensen & Caine, 2021). The results from the current study, however, showed most of the tourists did not detect the vipers when they were on the paths, and almost none of them could detect vipers, lying in the grass right next to the paths. This clearly contradicts the results of studies, conducted under controlled artificial circumstances (Öhman, Flykt & Esteves, 2001; LoBue & De Loache, 2008; LoBue & De Loache, 2011; Soares et al., 2014; Van Strien, Franken & Huijding, 2014; Van Strien & Isbell, 2017) or in a virtual simulation (Jensen & Caine, 2021), where people demonstrated increased snake detection ability. Using a somewhat similar natural approach, realistic models of *V. berus* (with a dorsal zig-zag stripe and size of 40 cm) were detected in less than 50% of the cases, in contrast to more unrealistic uniform models, which were registered much more often, with detection patterns also affected by individual body color and posture (Valkonen et al., 2020). Lock & Griffiths (2022) found that experienced surveyors detected 72% of *Natrix helvetica* (Lacépède, 1789) models in the field, whereas inexperienced surveyors detected between 53% and 58%. In the study of Albergoni et al. (2016) participants detected fewer than 50% of the models on the field, and detection probability depended on factors such as surveyor experience, whether they worked alone or in groups, and model placement. In each of these three studies, however, participants were actively searching for the snakes, in contrast to the current study, where participants were unaware of the vipers’ presence on the route, as well as of their involvement in the experiment. As stated by Jensen & Caine (2021), easily detectable objects might often become unnoticed when the person’s attention is focused on something else in the visual field. Indeed, while walking, most tourists looked at the surrounding landscape or talked to each other, rather than looking at their feet, which likely explains why many failed to notice the viper on the path. A high number of people would even step just next to the viper or step over it without detecting it. In a few cases, tourists even stepped on the viper without seeing it or saw it just after that. Even when tourists detected the viper, this typically occurred only at very close range, often just before stepping next to or directly in front of it. Such circumstances may increase the risk of snakebite incidents, as bites usually occur when people are in close proximity to a snake and are unaware of its presence or unable to avoid it Coelho et al. (2019). The results from the current study highlight the importance of research design and that future studies need to be based on real-life circumstances rather than in an artificial environment, in order to validate or reject the snake detection theory.

Interestingly, tourists detected *V. berus* more often than *V. ammodytes*, particularly in the case of adults. As both models and carcasses used for the two species were of similar size and coloration, these factors are unlikely to explain the observed difference in detectability. These differences might be attributed to distinct characteristics of the terrain and the habitat between the two sites. The path in the site of *V. berus* crosses through a relatively flat plateau area with few mountain peaks around it. In contrast, in the *V. ammodytes* site, the path crosses through a relatively steep and very scenic karst valley area, with many cliffs and vertical rock walls towering above it. It could be speculated that this more scenic landscape engages tourists’ attention more strongly than the relatively uniform environment of the *V. berus* site, leading them to pay less attention to the ground.

The results from the current study showed that, when on the paths, adult vipers were much more likely to be detected by tourists than juveniles, which is in agreement with results from previous studies on human detection on snake models (Albergoni et al., 2016; Lock & Griffiths, 2022). This is not surprising as the larger size of adult snakes makes them more conspicuous and easier to find than juveniles (Whitaker & Shine, 1999; Mazerolle et al., 2007; Steen, 2010; Durso & Seigel, 2015; Bauwens & Claus, 2018). Avian predators are also known to detect and prey on larger snakes more often than on smaller ones (Gil & Pleguezuelos, 2001; Niskanen & Mappes, 2005), although there are some exceptions from this pattern (Bittner, 2003).

### Tourists’ reactions towards detected vipers

Surprisingly, most tourists showed interest and no signs of fear, during their encounters with the viper models or carcasses. Even more surprising was the very small number of people reacting aggressively or showing strong fear during these encounters. Tourist’ predominant absence of fear towards the models might explain the very small number of aggressive reactions, as strong fear of snakes is usually the main driver of aggression towards them (Ballouard et al., 2013; García-López, Pacheco-Coronel & Gómez-Álvarez, 2017). Similar to the current study, predominantly positive behaviour towards snakes and reluctance about killing them were registered in several other surveys (Ballouard et al., 2012; Ballouard et al., 2013; Pandey et al., 2016; Keener-Eck, Morzillo & Christoffel, 2020; Valkonen et al., 2018). However, such results are the exception, as in most cases the negative attitudes and the willingness to kill snakes are the most common (Ballouard et al., 2013; Alves et al., 2014; García-López, Pacheco-Coronel & Gómez-Álvarez, 2017; Da Silva et al., 2021; Eid et al., 2021; Čubrić & Crnobrnja-Isailović, 2022; Kontsiotis, Rapti & Liordos, 2022; Pradhan & Yonle, 2021). The predominant interest and lack of fear in tourists in the current study might be due to the fact that vipers were immobile (*i.e.,* either models or dead individuals). Even though a dead snake can still cause animosity and aversion in people that are afraid of or simply do not like snakes, the levels of threat that it presents are not comparable to those of a living one. Because most people quickly recognized that the viper was not alive, this may have influenced their reactions, particularly the level of interest they showed, as they knew there was no risk of attack. However, initial reactions at the moment of detection are less likely to have been affected, as it is difficult in that brief instant to determine whether the snake is dead or simply motionless. Therefore, responses related to fear and aggression are likely less influenced by this aspect of the study design. It is noteworthy to say that among the tourists who visited this area during the course of this study, it could be that many had developed high environmental conservation awareness and respect towards nature and wildlife in general. This might further explain the small registered number of negative and aggressive reactions in the current study. Based on the author’s personal previous communications with local people and farmers from various villages in Bulgaria, the latter would be expected to express much more negative and aggressive reactions towards vipers and snakes in general. Similarly, in Nepal, negative attitudes towards snakes increased in agricultural fields and near the homes of the locals, and farmers and illiterate people were much more negative towards them than students and literate people (Pandey et al., 2016). So, further studies, following the current methodological approach, should focus on evaluating the attitudes and reactions of local people during their encounters with snakes.

As bigger snakes may be perceived as more intimidating than smaller ones by humans (Landová et al., 2012), the author’s expectations were that reactions towards the former will be stronger and more negative than towards the latter. However, there were no clearly distinct differences between most of the main reaction types towards juvenile and adult vipers. The only exceptions were evident in the categories No interest and Moderate fear with no interest, where tourists exhibited these reactions more often towards adult vipers than towards juveniles. These results might indicate that because adult vipers cause moderate fear in some of the tourists more often than juveniles do, these tourists tend to pass them by, without stopping to take a better look at them. However, as the results for the other reaction types were not that clearly distinct between adult and juvenile vipers, this notion cannot be fully confirmed.

### Species identification and additional information

Most tourists who tried to identify the species in the current study either identified it correctly or at least identified that the snake is venomous. In contrast, results from other studies show that most surveyed people generally cannot correctly identify the local snake species or they cannot distinguish between venomous and non-venomous species (Morrison et al., 1983; Ballouard et al., 2013; Pandey et al., 2016; Valkonen et al., 2018; Wolfe, Fleming & Bateman, 2020; Eid et al., 2021; Kontsiotis, Rapti & Liordos, 2022; Pradhan & Yonle, 2021), although some exceptions are also evident (Corbett et al., 2005; Alves et al., 2014; García-López, Pacheco-Coronel & Gómez-Álvarez, 2017). As *V. ammodytes* is a species with quite a distinctive morphology, due to the presence of the characteristic horn on its snout (Speybroeck et al., 2016), this might explain the high degree of correct identifications, as most of them were indeed made during the experiments with this species. In contrast, most misidentifications were made during the experiments with *V. berus*. Similarly, almost half of interviewed local people from Serbian villages were able to positively identify *V. ammodytes* (Čubrić & Crnobrnja-Isailović, 2022). Interestingly, most positive identifications in the current study were on the model of juvenile *V. ammodytes*, rather than on the adult carcass. If anything, this, together with the fact that most people did not understand that the models of both species were not real vipers, shows that their appearance was accurate. It should be noted, however, that the results might be prone to methodological bias, since information about the species identification could be gathered only from people who discussed the species affiliation of the snake between themselves. However, since most tourists either did not try to identify the snake, or at least did not discuss it between them, it is impossible to know if they would have made a correct or incorrect identification. Therefore, it is possible that tourists’ ability to correctly identify vipers in the current study might be overestimated.

## Conclusions

The results from this innovative approach to study the different aspects of human-snake interactions were highly encouraging and proved that the use of snake models can be very useful in such studies. In regard to evaluating people’s ability to detect vipers, this approach, recreating a natural human-snake encounter as close as possible, generated contrastingly different results from studies, conducted under controlled artificial circumstances. The current results clearly show that in future, studies on people’s ability to detect snakes should incorporate more natural approach to validate or reject the snake detection theory. In regard to evaluating people’s attitude towards vipers and their reactions when encountering them, this approach also provided a novel view on these aspects of human-snake interactions. Even though the fact that snakes are immobile and dead might cause some bias in people’s reactions, this approach is useful for detecting subconscious fear and aggression towards snakes as well as for assessing people’s ability to identify them. Therefore, in future, more studies on people’s perception of snakes should incorporate this natural approach.

##  Supplemental Information

10.7717/peerj.21420/supp-1 Supplemental Information 1 Raw data. Sheet 4, row 1, column C (Идентификация): Identification
Sheet 5: Reactions: rows 99, 150 and 194, column U (Комбинирано): Combined.

## Additional Information and Declarations

### Data Availability

The following information was supplied regarding data availability: The raw data is available in the Supplemental File.
